# Real-world comparative evaluation of immune-related toxicities in non-small cell lung cancer patients treated with immune-checkpoint inhibitors in Greece and Sweden

**DOI:** 10.3389/fimmu.2026.1904934

**Published:** 2026-08-10

**Authors:** Andreas V. Koulouris, Ioannis P. Trontzas, Ioannis A. Vathiotis, Maria Effrosyni Livanou, Marcus Skribek, Emmanouil Panagiotou, Athanasios Trimis, Simon Ekman, Georgios Tsakonas, Konstantinos N. Syrigos

**Affiliations:** 1 Medical Oncology, Thoracic Oncology Center, Karolinska University Hospital, Stockholm, Sweden; 2 Department of Oncology-Pathology, Karolinska Institutet, Stockholm, Sweden; 3 Medical Oncology, Third Department of Internal Medicine, Sotiria Hospital of Respiratory Diseases, National and Kapodistrian University of Athens, Athens, Greece; 4 Clinical Oncology, Thoracic Oncology Center, Karolinska University Hospital, Stockholm, Sweden; 5 Department of Medical Oncology, Bank of Cyprus Oncology Center, Nicosia, Cyprus; 6 Third Department of Internal Medicine, Sotiria Hospital of Respiratory Diseases; National and Kapodistrian University of Athens, Athens, Greece

**Keywords:** cross-national epidemiology, immune-related adverse events, immunotherapy, non-small cell lung cancer, real-world data, toxicity

## Abstract

**Background:**

Immune-related adverse events (irAEs) are common complications of immunotherapy, yet real-world evidence on how irAE patterns vary across patients from different geoenvironmental or genetic backgrounds remains limited. Observed differences in autoimmune disease prevalence across European regions motivated an exploratory comparison of irAEs in two large ICI-treated non-small cell lung cancer (NSCLC) cohorts.

**Methods:**

We conducted a retrospective study of two independently assembled cohorts of consecutive patients with advanced NSCLC treated with immunotherapy at specialized thoracic oncology departments in Southern Europe (Sotiria Thoracic Diseases Hospital of Athens, Greece; 2014-2023) and Northern Europe (Karolinska University Hospital, Sweden; 2015-2022). Clinical characteristics, treatment patterns, and irAEs (CTCAE v5.0) were harmonized into a unified dataset. Descriptive statistics were reported and compared between the two cohorts. Multivariable logistic regression evaluated the independent association between cohort and (i) any irAE, (ii) multiple irAEs, and (iii) grade≥3 irAEs.

**Results:**

Of 1,331 included patients (653 Greek, 678 Swedish), treatment patterns were largely similar. Overall, irAE incidence did not differ significantly, nor did the rate of multiple irAEs. However, hepatitis and nephritis were more common in the Swedish population (11.4% vs. 5.7%, *p* = 0.0002; and 4.0% vs. 0.5%, *p* < 0.0001, respectively). Severe irAEs (grade≥3) were more frequent in Sweden (21.9% vs. 10.4%; *p* < 0.0001). In the multivariable analysis, irAEs severity remained significantly more prominent in the Swedish cohort (OR:2.04, 95%CI:1.40-2.99).

**Conclusion:**

In this bi-national cohort, overall and multisystem irAE rates were similar, but severe and selected organ-specific toxicities were more frequent in the Swedish population. Geographic and host-related factors may modulate irAE severity and should inform risk-adapted toxicity surveillance.

## Graphical Abstract

**Figure:**
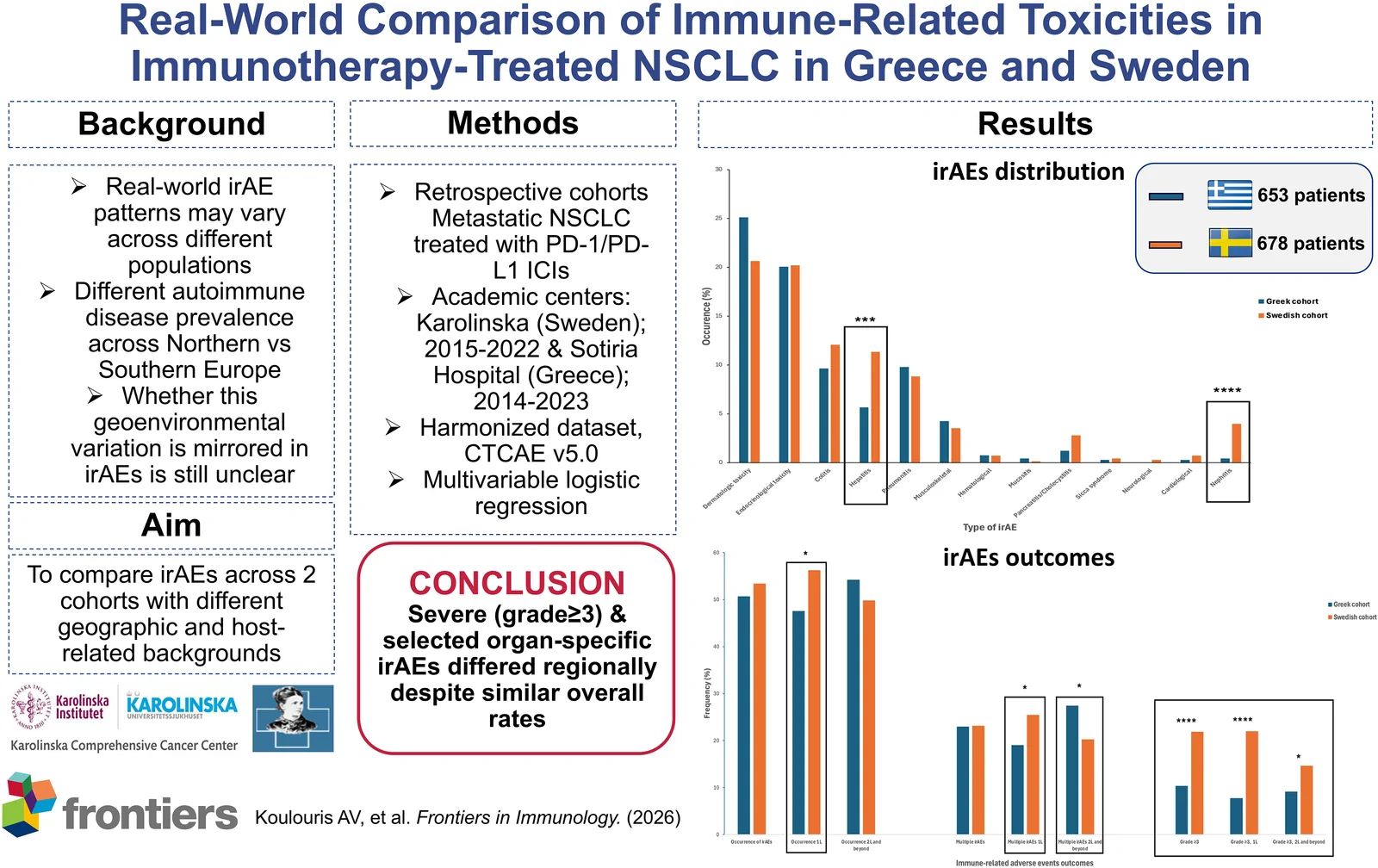

## Highlights

- Few available data on geographic variation in immune-related adverse events (irAEs) among patients receiving immune checkpoint inhibitors (ICIs).
- In 1,331 patients with advanced non-small cell lung cancer (NSCLC) from Sweden and Greece, severe (grade≥3) irAEs were more than twice as common in Sweden.
- The Swedish cohort remained independently associated with grade≥3 irAEs after multivariable adjustment (OR:2.04, 95%CI:1.40–2.99).
- Immune-related hepatitis and nephritis occurred significantly more frequently in the Swedish cohort.
- These findings support investigation of geoepidemiologic factors and risk-adapted toxicity surveillance strategies.

## 1 Introduction

Immune checkpoint inhibitors (ICIs) have reshaped the treatment landscape of advanced non-small cell lung cancer (NSCLC), improving survival across multiple clinical settings (1–5). By inhibiting immune checkpoint pathways, such as programmed cell death protein-1/programmed death-ligand 1 (PD-1/PD-L1), ICIs restore antitumor T-cell activity but may also disrupt peripheral tolerance, leading to immune-related adverse events (irAEs) (6). irAEs remain a major limitation of ICI-therapy, occurring in 30%-40% of NSCLC patients and leading to treatment discontinuation in 3%-12% of cases (7, 8). Dermatologic and endocrine toxicities are among the earliest and most common manifestations, but irAEs can involve virtually any organ system, with the potential for severe and life-threatening complications (9). Although the incidence and spectrum of irAEs are well described in clinical trials, far less is known about how their occurrence may vary across different populations or environmental contexts.

A growing body of evidence suggests that the prevalence of autoimmune diseases -conditions that share pathophysiologic features and clinical phenotypes with irAEs- demonstrates substantial geographic and climatic variation (10). Given that irAEs reflect dysregulated immune activation driven by ICIs, it is biologically plausible that host-related or geoenvironmental factors influencing autoimmunity may also modulate susceptibility to immunotherapy toxicity. Particularly, host-specific factors, such as germline immune-regulatory variants and gut microbiome composition, may influence susceptibility to irAEs, suggesting that population-level immune differences could also shape toxicity patterns (11). Yet despite the rapid global expansion of ICIs, real-world comparative data on irAEs across different populations remain limited and heterogeneous (12–14).

The aforementioned observations underscore an important knowledge gap: whether the incidence, severity, and organ-specific patterns of irAEs vary across populations exposed to different environmental, genetic, or geoepidemiologic backgrounds. Understanding such variation is clinically relevant, as it may inform individualized toxicity surveillance and management strategies in diverse health-care settings. In this context, we conducted a real-world comparative study in two European populations of patients with NSCLC exposed to contrasting geoenvironmental conditions -one at high latitude and one in Southern Europe. This study aims to address the limited evidence on geoepidemiologic patterns of irAEs and to provide insights into potential population-level modifiers of irAE risk.

## 2 Methods

### 2.1 Study population and data sources

This retrospective comparative study included patients with histologically confirmed, metastatic NSCLC who were treated with ICIs in two different institutions. Two independently assembled cohorts were analyzed. The Southern European cohort comprised all consecutive patients treated in the Oncology Unit, 3^rd^ Department of Internal Medicine, ‘Sotiria’ Hospital, National and Kapodistrian University of Athens, Greece, from 2014 to 2023. The Northern European cohort consisted of an all-comers population treated between 2015 and 2022 in the Thoracic Oncology Department of Karolinska University Hospital, Stockholm, Sweden.

**Figure 1:**
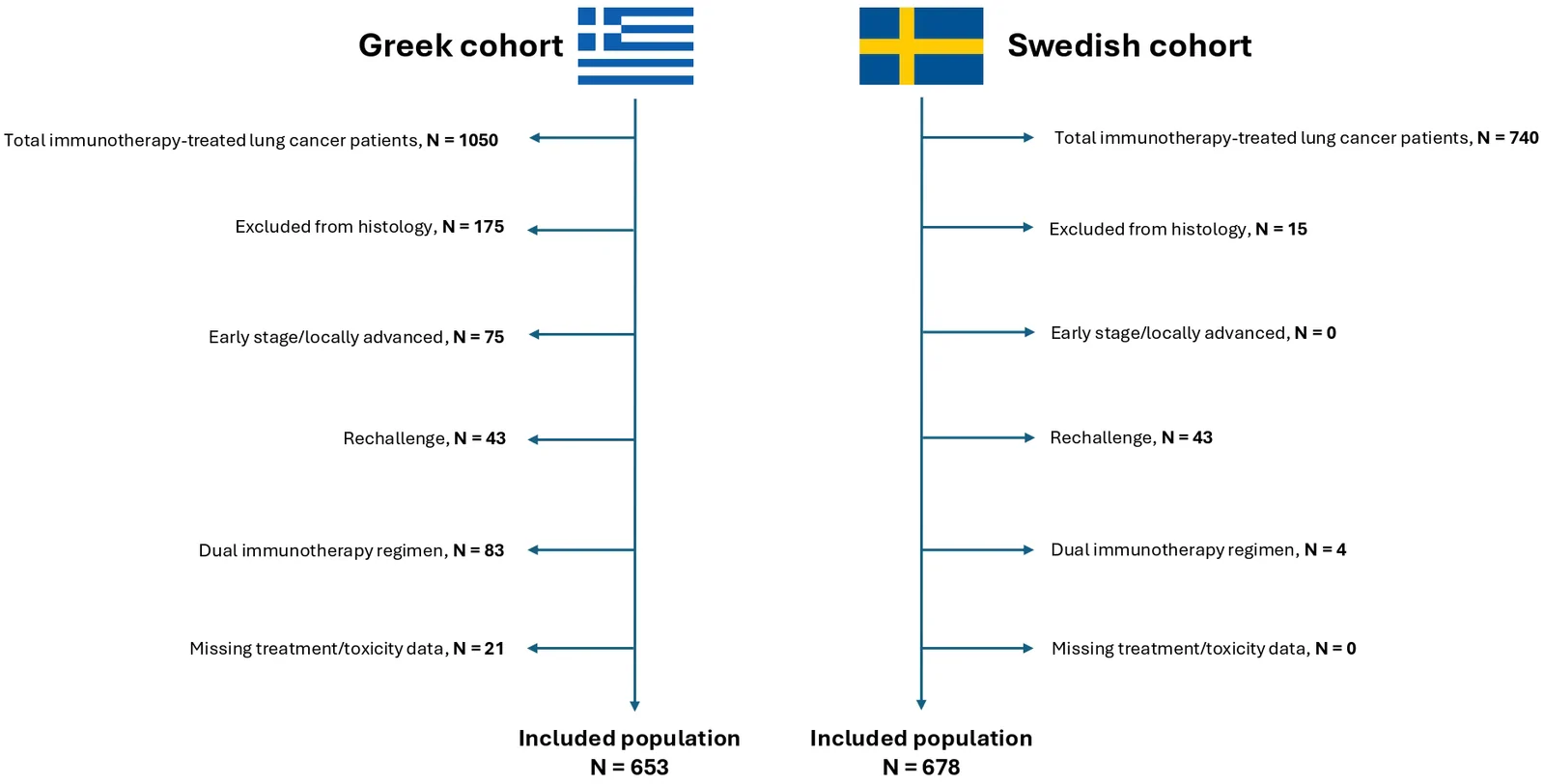
Flowchart of included cases from original institutional databases.

Eligibility criteria were consistent across both cohorts. Patients were included if they received at least one dose of ICIs for advanced/metastatic NSCLC. Individuals with early-stage disease receiving neoadjuvant, adjuvant, or consolidation ICIs regimens (e.g., PACIFIC protocol) were excluded (15). Cases of ICI rechallenge treatment were analyzed only for their first exposure to ICIs. After preliminary analysis, patients treated with dual ICIs combinations were also excluded due to a significant imbalance in their use between the two cohorts. Cases of neuroendocrine tumors or tumors with mixed neuroendocrine histology or NSCLC subtypes with distinct biological behavior from conventional non-squamous and squamous histologies (e.g., adenoid cystic carcinomas, mucoepidermoid carcinomas) were also excluded. Finally, cases with missing or insufficient data on toxicity outcomes or treatment characteristics were not considered for analysis (**Figure 1**).

### 2.2 Data collection

Data were retrospectively extracted from both electronic and paper-based medical records at participating centers in Greece and Sweden. To ensure comparability between populations, collected variables were restructured and harmonized into a standardized, homogeneous database template focused on outcomes of interest.

Baseline clinical and pathological characteristics included sex, age, Eastern Cooperative Oncology Group-performance status (ECOG-PS), autoimmune comorbidities, NSCLC histological subtype, disease stage at initiation of ICI therapy, PD-L1 expression (assessed by tumor proportion score, TPS), and genetic alterations. Treatment-related data included line of immunotherapy, ICI regimen (monotherapy or chemo-immunotherapy), specific ICI agent and target, and median duration of ICI exposure. Moreover, irAEs were documented in terms of overall frequency, affected organ system, occurrence of multisystem involvement (defined as ≥2systems affected), severity (grading per Common Terminology Criteria for Adverse Events, CTCAE, v.5.0), and the need for corticosteroids or other immunomodulatory interventions. Organ-system categories were predefined and captured as: dermatologic, endocrine, gastrointestinal (including colitis), hepatic (hepatitis), pulmonary (pneumonitis), musculoskeletal, hematologic, mucositis, pancreatitis/cholecystitis, sicca syndrome, neurologic, cardiac, and renal (nephritis) irAEs.

### 2.3 Outcomes

The primary outcome of the study was to compare the incidence and patterns of irAEs between the Southern and Northern European cohorts of patients treated with ICIs for advanced NSCLC. Specific outcomes assessed included the overall occurrence of irAEs, the frequency of high-grade events (grade≥3), the distribution of systems involved, the rate of multisystem involvement, and the need for immunosuppressive treatment. Distribution was assessed across the predefined organ-system categories described above. Additional comparisons were made based on treatment-related factors, including line of treatment, use of monotherapy versus chemo-immunotherapy, and PD-L1 expression levels.

### 2.4 Ethical considerations

All data were collected and processed in accordance with the Declaration of Helsinki. The study protocol was approved by the institutional review boards of the participating centers in Greece (approval no.: 22353/09-09-2025) and Sweden (approval no.: 2020-02636/2020-09-19). Study-specific informed consent was not required under the approvals granted by the relevant ethics committees. Consent and data-use procedures for the Greek cohort followed local institutional requirements. All patient data was de-identified and handled in accordance with applicable institutional policies and the General Data Protection Regulation (GDPR).

### 2.5 Statistical analysis

Descriptive statistics were used to summarize baseline characteristics, treatment-related variables, and irAEs. Categorical variables were expressed as frequencies and percentages, and they were compared using the chi-square test or Fisher’s exact test as appropriate. For continuous variables, normality of distribution was assessed using the Shapiro-Wilk test. Variables not following a normal distribution were reported as medians (range) and compared using the Mann-Whitney U test. Multivariable logistic regression was performed to estimate the independent association between treatment cohort (Southern European versus Northern European) and the risk of irAEs while adjusting for potential confounders. Separate models were constructed for three binary outcomes: any irAE, multiple irAEs (≥2systems involved), and grade≥3 irAEs. All multivariable models included treatment cohort, sex, age, ECOG-PS, autoimmune comorbidity, and line of immunotherapy as prespecified covariates selected *a priori* based on biological plausibility, clinical relevance to irAE risk, and key baseline imbalances between cohorts. Covariate selection was not based solely on statistical imbalance. Smoking status and histology were not included in the primary models because they are not consistently established as independent predictors of irAE risk, whereas ICI target was not modeled separately because of its strong correlation with treatment cohort and the low frequency of PD-L1 inhibitor use. An expanded multivariable sensitivity analysis including these additional baseline and treatment-related variables is provided in the Supplementary Material.

Model calibration was assessed using the Hosmer–Lemeshow goodness-of-fit test. All statistical tests were two-sided, with *p*-values<0.05 considered statistically significant. Analyses were conducted using GraphPad Prism version 10.4.1 (GraphPad Software, San Diego, CA, USA; available at: https://www.graphpad.com/). This study is reported in accordance with the Strengthening the Reporting of Observational studies in Epidemiology (STROBE) guidelines; the completed checklist is provided in the **Supplementary Material**.

## 3 Results

### 3.1 Baseline clinical and pathological characteristics

A total of 1,331 patients with advanced or metastatic NSCLC treated with ICIs were included in the study, comprising 653 patients in the Southern European cohort and 678 in the Northern European cohort. Significant differences were observed between the two populations across several baseline characteristics (**Table 1**). Patients in the Greek cohort were generally younger (*p* < 0.0001), more often male (*p* < 0.0001), and had a worse ECOG-PS (*p* < 0.0001), whereas patients in the Swedish cohort had a higher prevalence of autoimmune comorbidities (*p* < 0.0001). Smoking patterns also differed, with a substantially greater proportion of current smokers in the Greek cohort (*p* < 0.0001). No significant differences were observed in PD-L1 status between cohorts (*p* = 0.12). In contrast, molecular testing rates were markedly higher in the Swedish population (*p* < 0.0001). Among tested patients, *KRAS* mutations were more frequent in the Swedish cohort (*p* < 0.0001), whereas *TP53* (*p* < 0.01) and *STK11* (*p* < 0.0001) alterations were more common in the Greek population (**Supplementary Table 1**). No significant differences were observed in other actionable drivers.

**Table 1:** Baseline characteristics and treatment modalities of the two cohorts.

Characteristics	Greek cohort (%)	Swedish cohort (%)	*p*-value
Patients	653 (100.0)	678 (100.0)	
Gender (male)	497 (76.1)	302 (44.5)	<0.0001
Age*	68.0 (37-92)	71 (29-87)	<0.0001
Poor ECOG-PS (≥2)	188 (29.9)	96 (14.2)	<0.0001
Never smokers	25 (4.1)	56 (8.3)	0.0002
Current smokers	405 (66.9)	241 (35.6)	<0.0001
Autoimmune comorbidities	20 (3.2)	62 (9.1)	<0.0001
Histological type (adenocarcinoma)	405 (62.0)	481 (70.9)	<0.0001
PD-L1 positive (>1%, TPS)	385 (70.6)	422 (72.5)	0.12
PD-L1 high (≥50%)	212 (38.9)	235 (40.4)	0.35
Line of treatment			
First line	347 (53.1)	377 (55.6)	0.26
Second line	223 (34.2)	221 (35.6)	0.55
Third or later lines	83 (12.7)	80 (11.8)	0.67
Immunotherapy exposure time (months)*			
First line	4.2 (0-60.3)	4.9 (0-32.4)	0.15
Second line	2.3 (0-42.9)	3.5 (0-64.2)	0.27
Third or later lines	2.3 (0-29.5)	2.8 (0-28.2)	0.58
Immunotherapy regimen			
Monotherapy	400 (61.3)	418 (61.6)	0.93
Chemo-immunotherapy	253 (38.7)	260 (38.4)	
Immunotherapy targets			
PD-1	637 (97.6)	631 (93.1)	<0.001
PD-L1	16 (2.4)	47 (6.9)	
Immunotherapy agents			
Pembrolizumab	412 (63.1)	468 (69.0)	
Nivolumab	224 (34.3)	159 (23.5)	
Atezolizumab	16 (2.54)	45 (6.6)	
Cemiplimab	1 (0.2)	4 (0.6)	
Durvalumab	0 (0)	2 (0.3)	

*Median (range).

ECOG-PS, Eastern Cooperative Oncology Group-performance status; ICI, Immune checkpoint inhibitor; TPS, Tumor proportion score.

### 3.2 Treatment modalities

Treatment patterns across the two cohorts were largely similar in terms of line of therapy and regimen type (**Table 1**). The proportion of patients receiving ICI monotherapy versus chemo-immunotherapy was nearly identical (*p* = 0.93). Median immunotherapy exposure times did not differ meaningfully (*p* = 0.18). However, notable differences were observed in the choice of ICI target and agent. PD-1 inhibitors were more commonly used in the cohort from Southern Europe, while PD-L1 inhibitors were more frequently used in the Northern European cohort (*p* < 0.0001).

### 3.3 Comparative incidence and severity of irAEs

**Figure 2:**
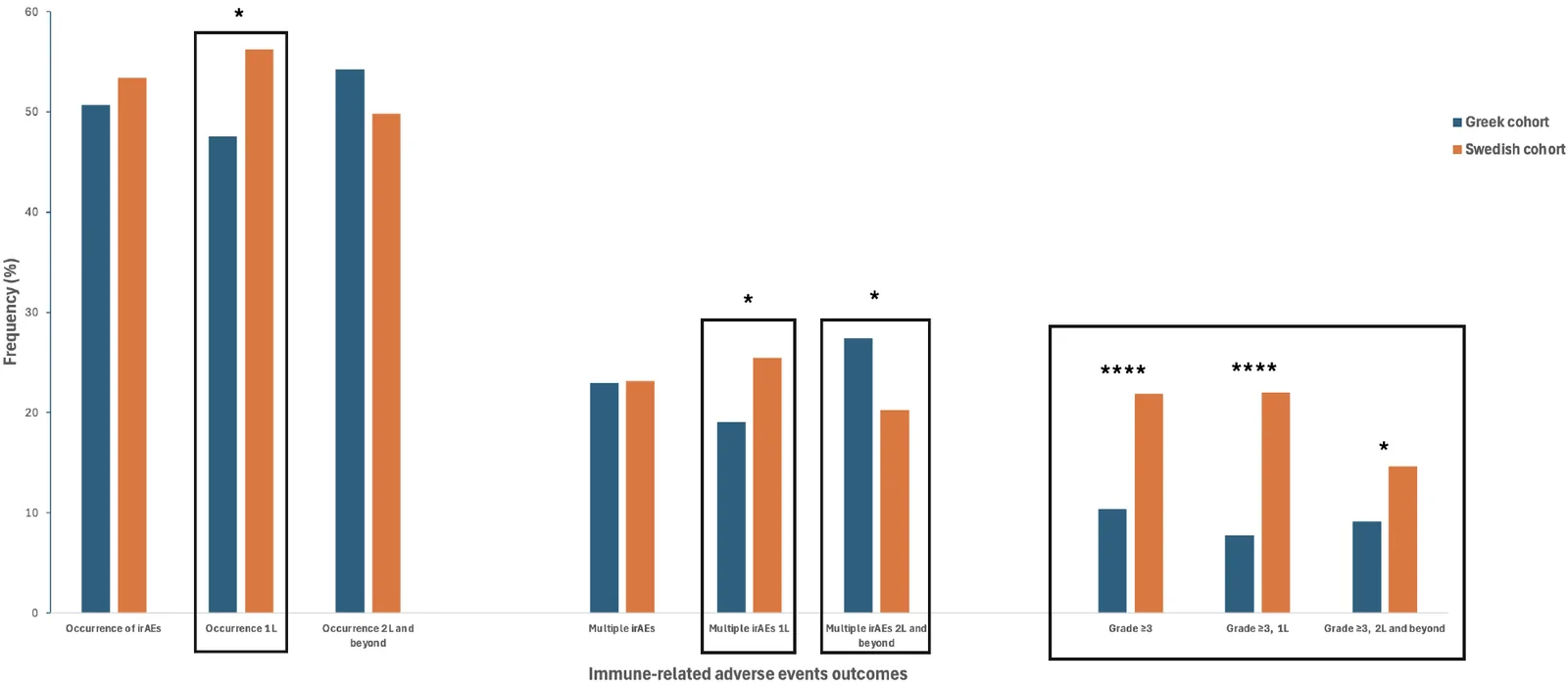
Proportions of major irAE outcomes in the Greek and Swedish cohorts, including overall irAEs, multiple irAEs, and grade ≥3 irAEs. In the first-line setting, both any irAE and multiple irAEs occurred more frequently in Swedish patients, whereas no differences were observed in later-line therapy. Severe irAEs (grade ≥3) were consistently more common in the Swedish cohort, both in first-line and subsequent treatment lines. irAEs, immune-related adverse event. Statistical significance: *p<0.05; ****p<0.0001.

The overall incidence of irAEs was comparable between the two cohorts, occurring in 50.7% of patients in the Greek group and 53.4% in the Swedish group (*p* = 0.32) (**Table 2**; **Figure 2**). However, when stratified by line of treatment, irAEs were significantly more frequent among Swedish patients receiving first-line ICIs (56.2% vs. 47.6%, *p* = 0.02), and among those with high PD-L1 expression (60.4% vs. 46.7%, *p* < 0.01), whereas no statistically significant differences were seen in later-line therapy or in patients with lower PD-L1 levels. irAEs were more frequent among Swedish patients receiving ICI monotherapy in the first-line setting (58.6% vs. 43.6%, *p* = 0.02), whereas rates among those receiving chemo-immunotherapy did not differ significantly (*p* = 0.18). Rates of multiple irAEs were also similar overall, though Swedish patients showed higher frequencies in first-line treatment, and Greek patients in later-line settings.

**Table 2:** Comparison of irAE outcomes between the two cohorts.

irAEs	Greek cohort (%)	Swedish cohort (%)	*p*-value
Patients	653 (100.0)	678 (100.0)	
irAEs occurrence (overall)			
**irAEs occurrence (overall)**	**331 (50.7)**	**362 (53.4)**	**0.32**
irAEs occurrence per line of treatment			
First line	165 (47.6)	212 (56.2)	0.02
Second line and beyond	166 (54.3)	150 (49.8)	0.28
irAEs occurrence per PD-L1 status			
PD-L1 <50%	168 (47.6)	171 (49.1)	0.68
PD-L1 high (≥50%)	99 (46.7)	142 (60.4)	<0.01
irAEs occurrence per ICI regimen (1L included)			
ICI monotherapy	48 (43.6)	95 (58.6)	0.02
Chemo-immunotherapy	117 (48.2)	117 (54.4)	0.18
Multiple irAEs (overall)			
**Multiple irAEs (overall)**	**150 (23.0)**	**157 (23.2)**	**0.94**
Multiple irAEs per line of treatment			
First line	66 (19.0)	96 (25.5)	0.04
Second line and beyond	84 (27.5)	61 (20.3)	0.04
Multiple irAEs per PD-L1 status			
PD-L1 <50%	73 (20.7)	78 (22.4)	0.58
PD-L1 high (≥50%)	39 (18.4)	61 (26.0)	0.06
Multiple irAEs per ICI regimen (1L included)			
ICI monotherapy	18 (16.4)	43 (26.5)	<0.0001
Chemo-immunotherapy	48 (19.8)	23 (24.7)	0.21
Severity of irAEs			
Grade ≥3 (overall)	55 (10.4)	127 (21.9)	<0.0001
Grade ≥3 (1L)	27 (7.8)	83 (22.0)	<0.0001
Grade ≥3 (2L and beyond)	28 (9.2)	44 (14.6)	0.04
Corticosteroids/immunomodulators	166 (25.4)	174 (25.7)	>0.99
Death	3 (0.57)	4 (0.69)	>0.99

Bold p-values indicate statistically significant differences between cohorts (p<0.05). ICI, immune checkpoint inhibitor; irAEs, immune-related adverse events; L, line of treatment.

Of note, severe irAEs (grade≥3) occurred more than twice as often in the Northern European cohort compared to the South European cohort (21.9% vs. 10.4%; *p* < 0.0001). The severity was consistent both in the first line (7.8% vs. 22.0%, *p* < 0.0001) and in second-line or later (9.2% vs. 14.6%, *p* = 0.04).

### 3.4 Distribution of irAEs per organ system

**Figure 3:**
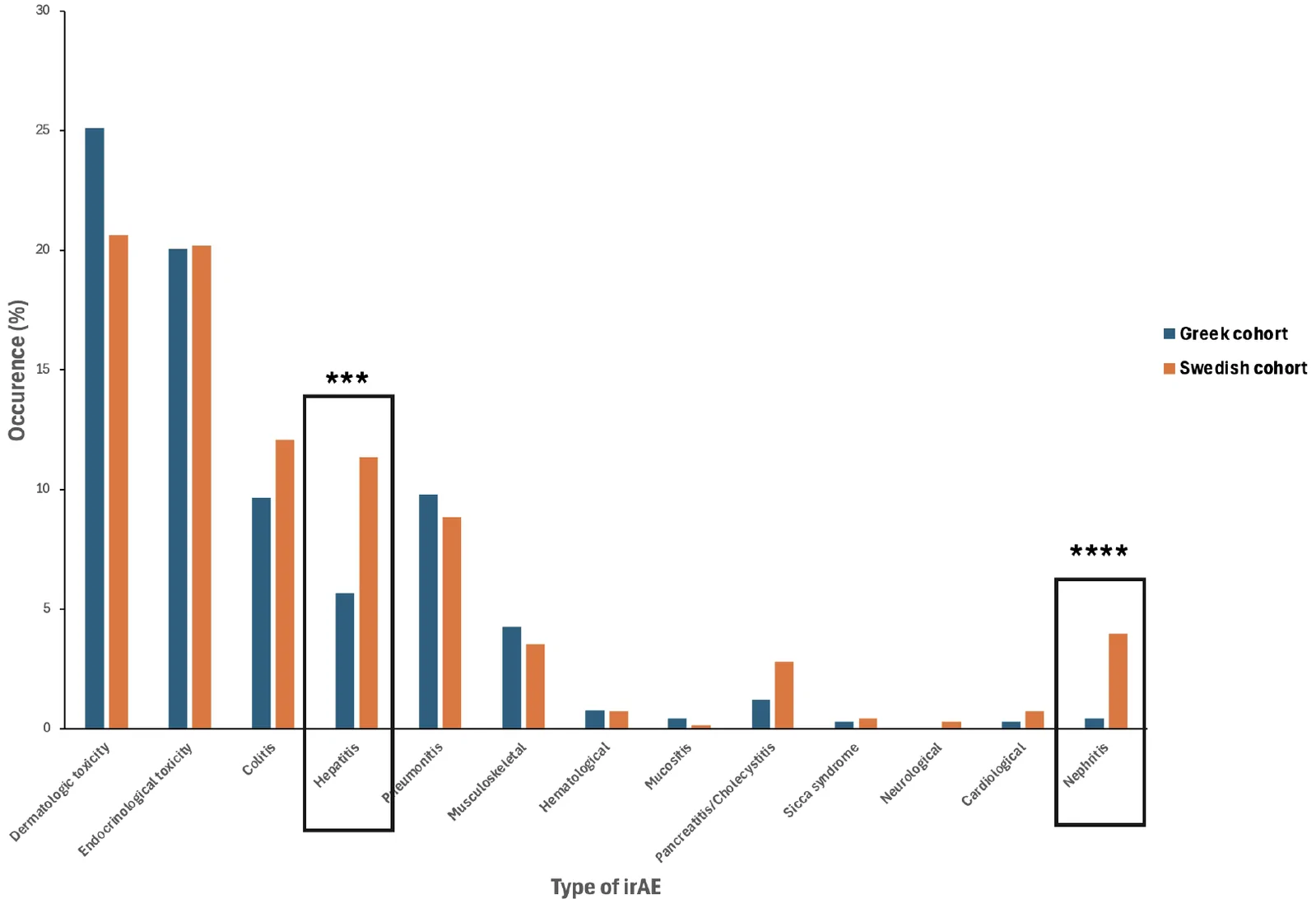
Distribution of irAEs events by affected organ system in the Greek and Swedish cohorts. Hepatitis and nephritis were significantly more frequent in the Swedish cohort, whereas dermatologic toxicities appeared more common among Greek patients, although this difference was not statistically significant. Other organ-specific irAEs occurred at broadly similar frequencies across cohorts. irAEs, immune-related adverse event. Statistical significance: ***p<0.001; ****p<0.0001.

Patterns of organ involvement differed between cohorts (**Figure 3**). In the Swedish population, the frequencies of hepatitis and nephritis were significantly higher (11.4% vs. 5.7%, *p* = 0.0002; and 4.0% vs. 0.5%, *p* < 0.0001, respectively). Dermatologic events appeared somewhat more frequent in the population from Southern Europe (25.1% vs. 20.6%), although this difference did not reach statistical significance (*p* = 0.052). Endocrine, pulmonary, gastrointestinal, and musculoskeletal toxicities occurred at similar rates. The predominant irAE types varied by treatment line in both cohorts (**Supplementary Figure 1**), with immune-related dermatitis, endocrinopathies, and colitis remaining among the most frequent across settings.

### 3.5 Multivariable analysis for irAEs outcomes

**Figure 4:**
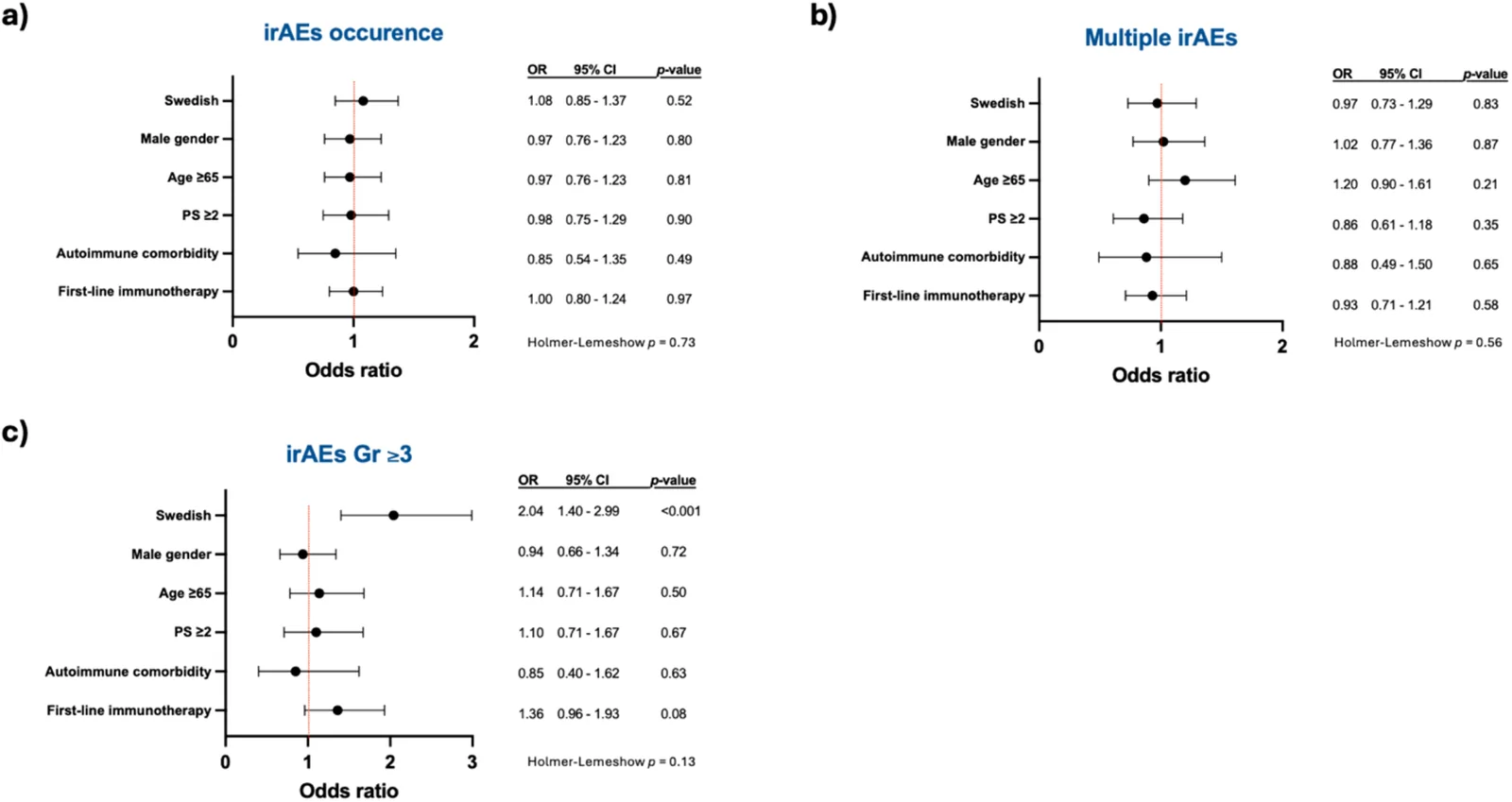
Multivariable logistic regression analyses showing adjusted odds ratios for **(a)** occurrence of any irAE, **(b)** occurrence of multiple irAEs, and **(c)** occurrence of grade ≥3 irAEs. Treatment cohort was not independently associated with the risk of overall or multiple irAEs; however, Swedish patients had a significantly higher adjusted risk of severe irAEs (grade ≥3). irAEs, immune-related adverse event; PS, Performance status; Gr, Grade.

After adjusting for potential confounders, cohort (Northern Europe vs. Southern Europe) was not associated with the risk of overall irAE occurrence or the development of multiple irAEs (**Figure 4**; **Supplementary Figure 2**; **Supplementary Table 2**). However, the treatment in the Swedish cohort remained a strong independent predictor of severe irAEs with more than a twofold increased risk compared to the Greek cohort (OR: 2.04, 95%CI: 1.40–2.99; *p* < 0.001). Model calibration was acceptable based on the Hosmer–Lemeshow goodness-of-fit (overall irAEs occurrence model *p* = 0.73, multiple irAEs model *p* = 0.56, and grade≥3 irAEs model *p* = 0.13). An expanded multivariable sensitivity analysis, including additional baseline and treatment-related covariates, yielded consistent results and is provided in **Supplementary Table 2** and **Supplementary Figure 2**.

## 4 Discussion

In this bi-national real-world study, we compared the incidence, severity, and distribution patterns of irAEs between two large European cohorts of NSCLC patients, exposed to contrasting geoenvironmental backgrounds. This comparative analysis revealed clinically meaningful differences in the pattern and severity of irAEs, supporting the hypothesis that geoepidemiologic or host-related factors may modulate immune toxicity in IC-treated patients.

The most striking finding was the consistently higher rate of severe irAEs in the cohort from Northern Europe, occurring more than twice as often as in the cohort from Southern Europe. This association remained robust after adjustment for measured demographic, clinical, and treatment-related covariates, including in the expanded sensitivity analysis, suggesting that the increased risk of grade ≥3 toxicity was not explained by the baseline factors included in the models. Severe irAEs are thought to reflect heightened or dysregulated immune activation, and their reproducible predominance in the Swedish population aligns with longstanding epidemiologic data showing higher burdens of autoimmune and inflammatory diseases at higher latitudes (16–22). This pattern strengthens the possibility that population-level immunologic backgrounds may influence the intensity of ICI-related immune activation.

Several autoimmune disorders, including sarcoidosis, systemic lupus erythematosus, autoimmune hepatitis, and rheumatoid arthritis, exhibit remarkable variation across ethnic, regional, and climatic gradients (16–22). One illustrative example is sarcoidosis, which shows among the highest global yearly incidence in Scandinavian countries (11.5/100,000 versus 0.5-1.3/100,000 persons in East Asia), with prevalence estimates reaching 160/100,000 in Sweden, compared to substantially lower rates in southern Europe and Asia (16–19). Recent geoepidemiological analyses further highlight strong correlations between lower ambient temperatures, increasing latitude, and higher prevalence of autoimmune diseases such as type 1 diabetes, rheumatoid arthritis, psoriasis, and inflammatory bowel disease (23). These gradients are thought to reflect complex interactions between genetic predisposition, environmental triggers, sunlight and vitamin D exposure, pathogen burden, and lifestyle patterns, all of which contribute to immune regulation and autoimmunity (23, 24). Such population-level differences in immune predisposition raise the question of whether the same factors might influence susceptibility to irAEs.

A second key observation was the early emergence of toxicity in the Swedish cohort, most notably during first-line therapy. Swedish patients receiving first-line ICI developed irAEs at significantly higher rates than Greek patients, whereas later-line toxicity rates converged. The fact that these differences were most pronounced in first-line therapy suggests that early immune activation may reveal underlying host-related susceptibility, supporting a geoepidemiologic rather than treatment-driven explanation.

Organ-specific patterns also differed between cohorts. Hepatitis and nephritis -both relatively uncommon but clinically impactful irAEs- were significantly more frequent in Sweden. Immune-related hepatitis was approximately twice as common in Swedish patients as in Greek patients (11.4% versus 5.7%), while nephritis was exceedingly rare in the Greek cohort (0.5%) yet eightfold more frequent in the Swedish cohort (4.0%). Because renal toxicity is unlikely to be missed in either setting (routine measurement of creatinine and liver enzymes is universal), this difference is unlikely to reflect surveillance bias. On the contrary, it may suggest distinct underlying immune programming affecting organ-specific susceptibility, although this hypothesis requires further investigation. Notably, population-based and biopsy-registry data for autoimmune kidney diseases, such as IgA nephropathy, show higher frequencies in northern European countries, with 40.6% of biopsy-proven glomerulonephritides in Sweden and 50.7% in northern Germany, compared to 35.2% in Italy (25–27). Incidence estimates demonstrate a similar north–south gradient, with higher rates in Sweden(1.27/100,000/year) and Germany(1.72) than in Spain(0.34) or Italy(0.84), which is consistent with a broader latitude-related gradient in renal autoimmunity, albeit with important methodological caveats (27).

In contrast, dermatologic irAEs were numerically more frequent in the Greek cohort. However, this difference did not reach statistical significance and should therefore be interpreted cautiously. Chronic ultraviolet exposure has been shown to modulate cutaneous immune responses through repeated antigenic stimulation, alterations in local immune cell composition, and changes in skin barrier function, providing a biologically plausible framework for regional variation in dermatologic immune toxicity (28).

These observations align with prior reports suggesting geographic heterogeneity in immunotherapy outcomes, although it has been incompletely characterized, particularly with respect to toxicity. A large meta-analysis of phase III trials demonstrated regional differences in the magnitude of benefit from ICIs, with patients from North America and Europe deriving greater overall survival benefit than those from Asia, and notable variation according to ICI class and line of therapy (12). Nevertheless, the region-driven impact on ICI efficacy remains heterogeneous across meta-analyses, with some updated NSCLC-specific data suggesting even greater benefit in East Asian than non-East Asian populations (29). In parallel, population-specific differences in irAEs profiles have been documented, including higher rates of pulmonary and hepatic irAEs in certain regions, potentially reflecting variation in host immune background, viral exposure, or genetic susceptibility (30, 31). However, most available data focus on broad continental comparisons and efficacy endpoints, with limited resolution within Europe and scarce real-world analyses of toxicity patterns. In this context, our study extends the current literature by demonstrating clinically meaningful differences in the severity, timing, and organ-specific distribution of irAEs between two European populations at different latitudes treated under comparable clinical conditions.

Several biological mechanisms may underlie the observed geographic heterogeneity in irAEs. ICIs disrupt peripheral tolerance, unmasking pre-existing immune reactivity that varies across individuals and populations (11). Longstanding epidemiologic data demonstrate strong latitude-dependent gradients in autoimmune disease prevalence, implicating environmental modifiers of immune homeostasis rather than treatment-related factors alone. Ultraviolet exposure and vitamin D signaling play crucial immunoregulatory roles by influencing antigen presentation, cytokine balance, and T-cell tolerance (32, 33). Lower ambient ultraviolet exposure at higher latitudes may predispose to heightened immune activation upon ICIs (32). Environmental factors varying by season and latitude, including infections, microbiome perturbations, and photoperiod-related immune rhythms, can influence both the timing and phenotype of irAEs (34). Geographic variation in gut microbiota composition represents a plausible additional modifier of irAEs. The microbiome is a key regulator of systemic immune tone and has been consistently linked to both the efficacy and toxicity of ICIs (35, 36). Dysbiosis can amplify immune activation and predispose to irAEs, including colitis and hepatitis (35–37). Population-level differences in dietary patterns, antibiotic exposure, infections, and baseline microbiome structure across Europe may therefore contribute to distinct immune setpoints at treatment initiation, influencing the timing, severity and organ-specific patterns of irAEs observed in this study (34, 36).

From a clinical standpoint, these findings have potential implications for risk-adapted toxicity surveillance in ICI-treated patients. The higher frequency, earlier onset, and greater severity of irAEs observed in the high-latitude cohort suggest that baseline population-level factors may help identify patients at increased risk for clinically significant toxicity. In such settings, closer monitoring during early treatment cycles, lower thresholds for laboratory surveillance, and heightened clinical vigilance -particularly for hepatic and renal toxicities- may be warranted. While these results do not support treatment modification at this stage, they raise the possibility that future strategies incorporating baseline risk stratification could inform more individualized approaches to immunotherapy delivery, including optimized dosing schedules or treatment de-escalation. In line with recent irAE research, prospective organ-specific cohorts with standardized diagnostic definitions and integrated biospecimen collection, including genomic, human leukocyte antigen (HLA), microbiome, and environmental data, may refine toxicity prediction and risk-adapted management (38). Prospective studies are also required to determine whether tailoring monitoring or treatment strategies based on geographic or host-related risk profiles can optimize safety without compromising efficacy.

Several design features strengthen the interpretability of these findings. Both cohorts included exclusively patients with advanced NSCLC, minimizing heterogeneity related to disease stage. The cohort sizes were nearly identical; data were independently curated and subsequently harmonized using uniform definitions and toxicity grading, and the enrollment periods overlapped for most of their duration (2015–2022). Although PD-L1 inhibitors were used slightly more frequently in the Swedish cohort than in the Greek cohort (6.9% versus 2.4%, respectively), overall treatment patterns were broadly similar. The Swedish cohort represents an all-comers population within a universal health-care system, whereas the Greek cohort reflects a high-volume specialized thoracic oncology center. Together, these complementary settings reduce the likelihood that the observed differences are driven solely by institutional practice patterns.

Nonetheless, limitations must be acknowledged. The retrospective design introduces potential information bias, although this was mitigated through standardized data harmonization and uniform CTCAE v5.0 grading. Missing data exclusions were minimal, affecting 21 of the 674 otherwise eligible Greek patients after preceding exclusion steps; no patients were excluded for missing data in the Swedish cohort. Retrospective attribution and grading of irAEs may have led to under- or overestimation of lower-grade events. Each cohort was derived from a single institution, which may limit generalizability beyond these settings. Although the enrollment periods overlapped for most of their duration, they were not identical, and temporal differences in ICI availability, regulatory approvals, or clinical practice cannot be entirely excluded. Dual checkpoint blockade was excluded, as its limited use in Sweden during the study period would have introduced a major imbalance; however, this restricts evaluation of combination-associated toxicities. Differences in the timing of irAE occurrence across treatment lines may also reflect exposure time and competing risks, as patients experiencing early severe toxicity may discontinue treatment sooner, thereby influencing later-line toxicity comparisons. Despite adjustment for prespecified clinical confounders (e.g., ECOG-PS, autoimmune comorbidity), residual confounding from unmeasured host factors, such as ethnic or genetic ancestry and environmental factors, such as prior infections, antibiotic exposure, vitamin D status, body mass index, or socioeconomic variables, cannot be excluded. Environmental, genomic, and microbiome data were not available, and therefore, the mechanistic basis of observed differences remains inferential. Although multivariable logistic regression was selected as the primary adjustment strategy to preserve the full analytic cohort, propensity score–based approaches were not performed and may represent useful complementary sensitivity analyses in future larger, multicenter datasets. Finally, although both centers used routine laboratory monitoring, subtle differences in clinical thresholds for ICI interruption or referral cannot be fully ruled out.

## 5 Conclusion

In conclusion, this bi-national real-world analysis suggests that irAEs are not uniform across European populations. Patients treated in a high-latitude setting experienced earlier onset, greater severity, and distinct organ-specific toxicity patterns, particularly involving hepatic and renal irAEs, compared to a southern European cohort. These consistent differences support the hypothesis that geoepidemiologic and host-related factors associated with latitude-linked autoimmunity may also modulate immune responsiveness during ICIs. Future prospective studies integrating genomic, environmental, and microbiome data are needed to define the biological basis of these observations and to inform risk-adapted toxicity surveillance strategies.

## Data Availability

The original contributions presented in the study are included in the article/**Supplementary Material**. Further inquiries can be directed to the corresponding author.
