# Supplementary material for: Real-world comparative evaluation of immune-related toxicities in non-small cell lung cancer patients treated with immune-checkpoint inhibitors in Greece and Sweden
Source: Front Immunol. 2026 Aug 10;17:1904934. doi: 10.3389/fimmu.2026.1904934 (PMC13500524; doi:10.3389/fimmu.2026.1904934)
Supplement: Supplementary file 1 [file Image1.pdf]

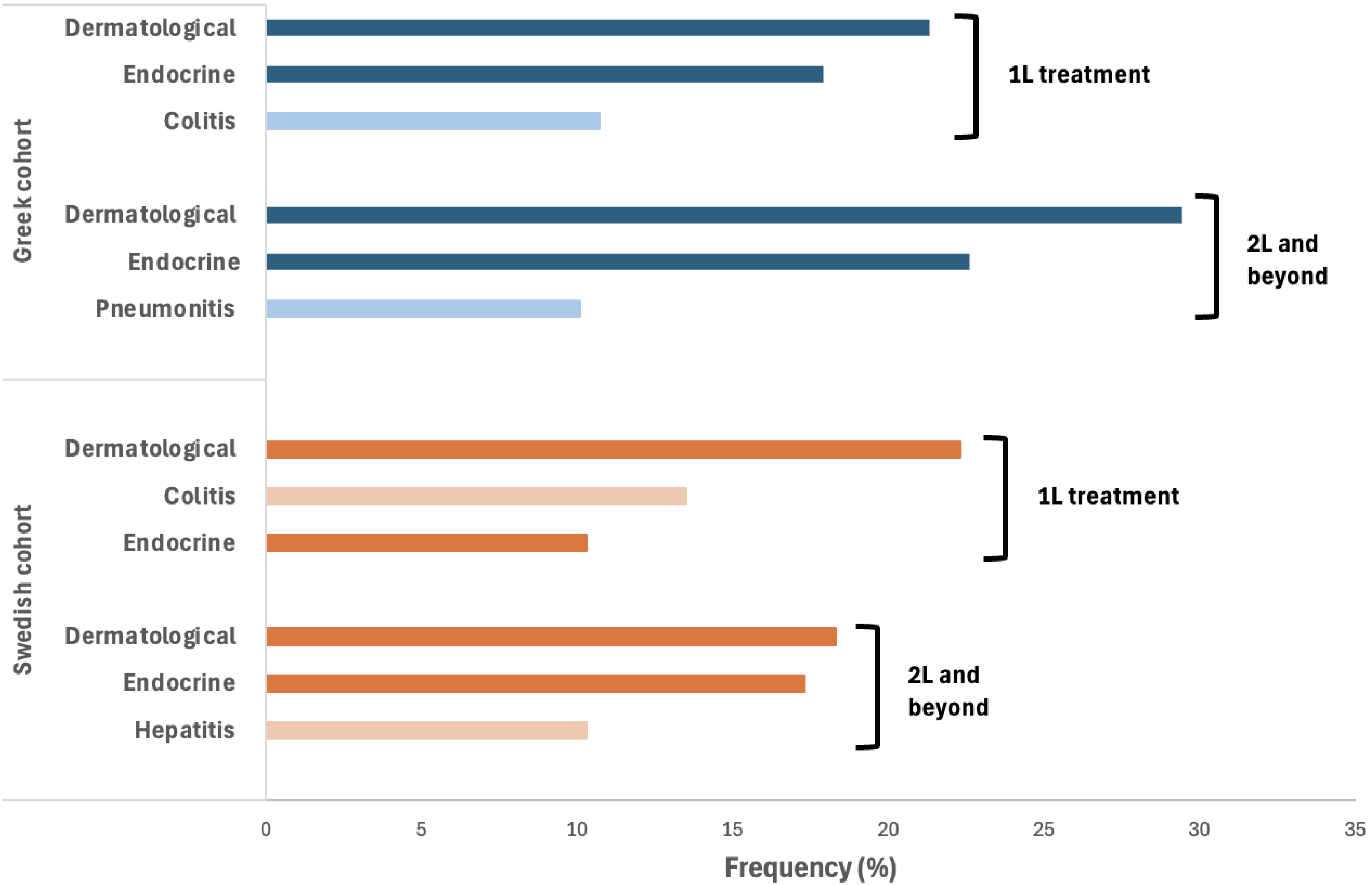
Greek cohort
Dermatological
Endocrine
Colitis
1L treatment
Dermatological
Endocrine
Pneumonitis
2L and beyond
Swedish cohort
Dermatological
Colitis
Endocrine
1L treatment
Dermatological
Endocrine
Hepatitis
2L and beyond
0
5
10
15
20
25
30
35
Frequency (%)
