# Supplementary material for: Real-world comparative evaluation of immune-related toxicities in non-small cell lung cancer patients treated with immune-checkpoint inhibitors in Greece and Sweden
Source: Front Immunol. 2026 Aug 10;17:1904934. doi: 10.3389/fimmu.2026.1904934 (PMC13500524; doi:10.3389/fimmu.2026.1904934)
Supplement: Supplementary file 2 [file Image2.pdf]

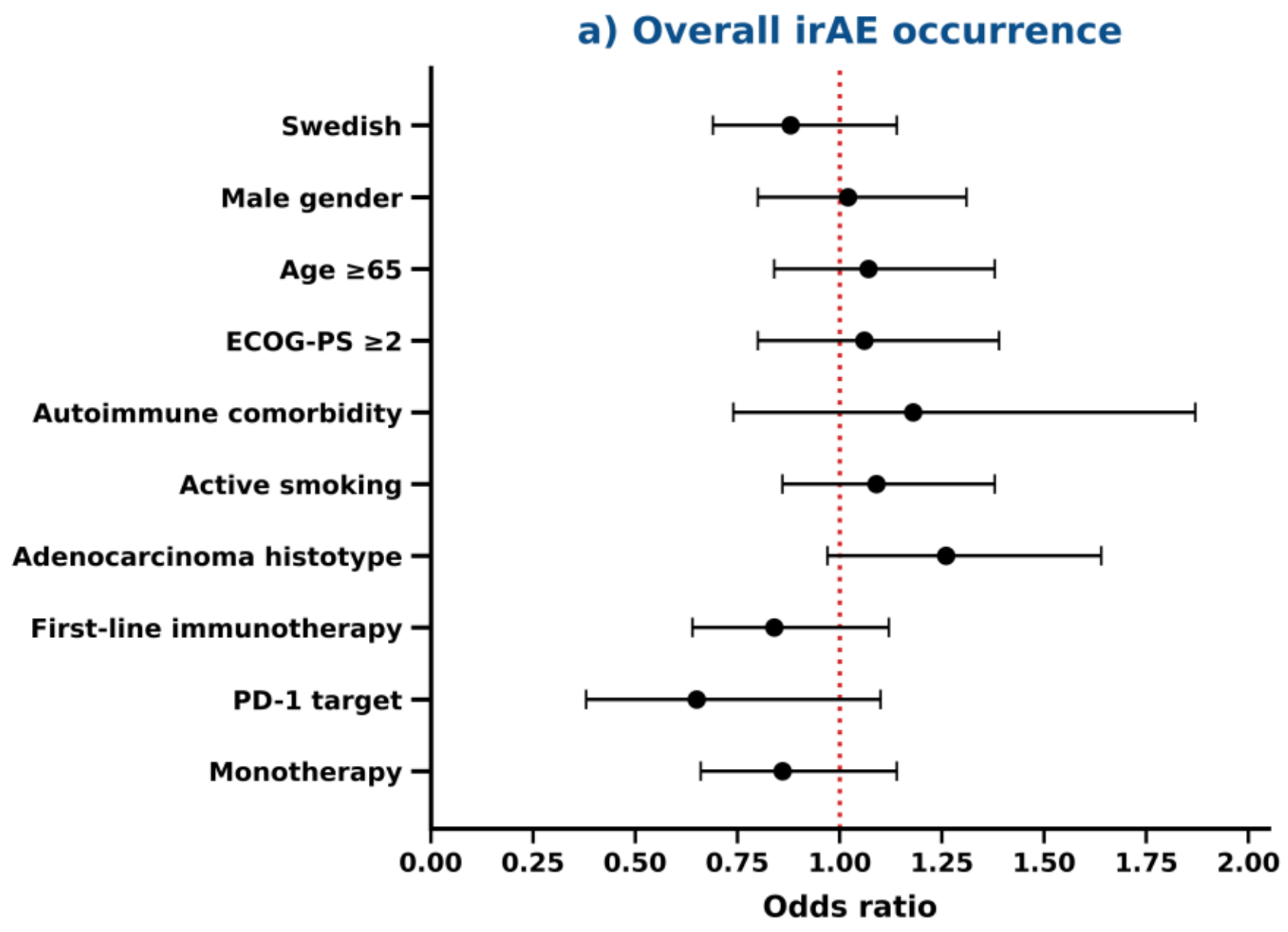

OR	95% CI	*p*-value
0.88	0.69 - 1.14	0.34
1.02	0.80 - 1.31	0.85
1.07	0.84 - 1.38	0.57
1.06	0.80 - 1.39	0.70
1.18	0.74 - 1.87	0.49
1.09	0.86 - 1.38	0.49
1.26	0.97 - 1.64	0.09
0.84	0.64 - 1.12	0.24
0.65	0.38 - 1.10	0.11
0.86	0.66 - 1.14	0.29

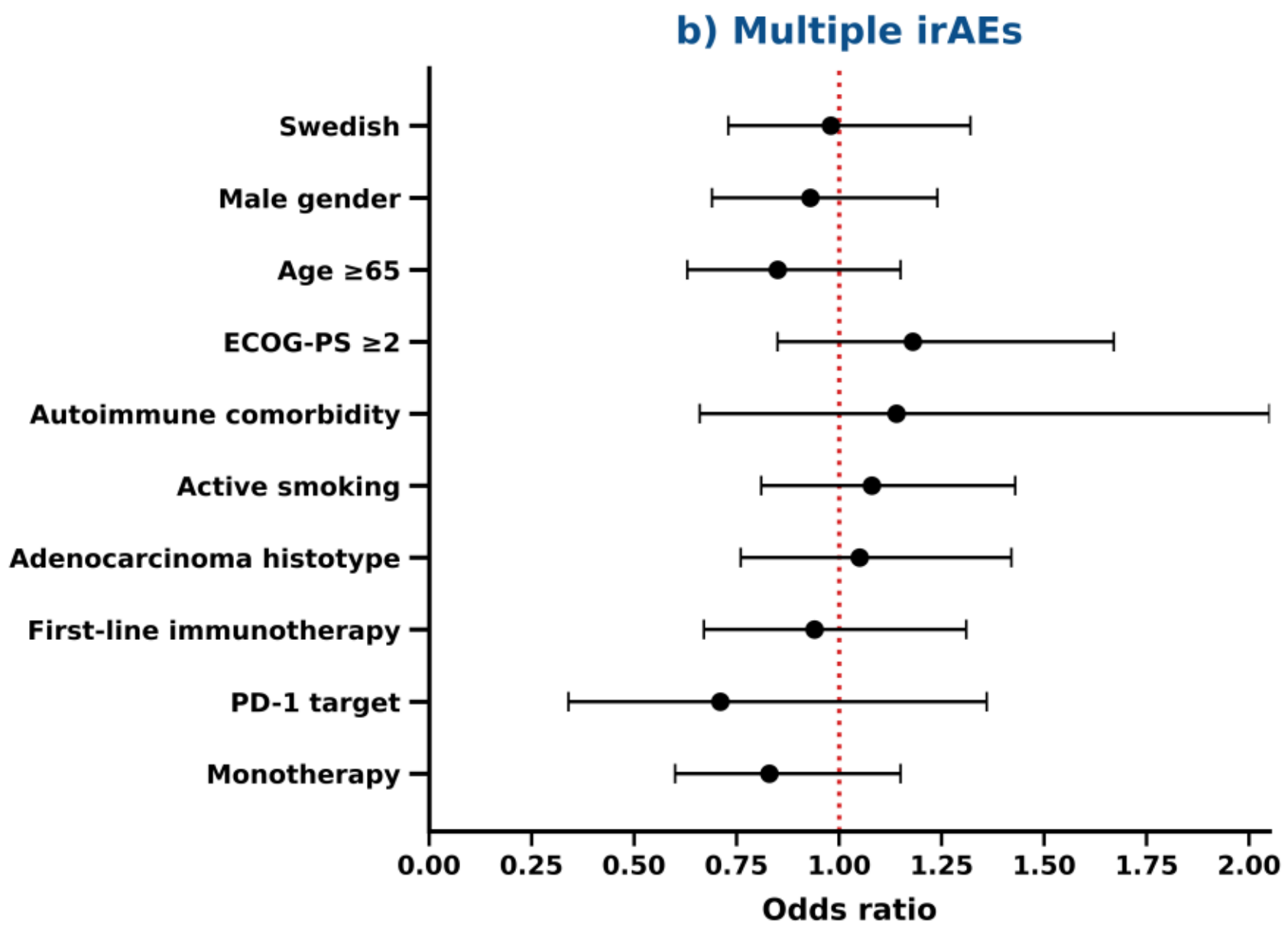

OR	95% CI	*p*-value
0.98	0.73 - 1.32	0.91
0.93	0.69 - 1.24	0.60
0.85	0.63 - 1.15	0.29
1.18	0.85 - 1.67	0.32
1.14	0.66 - 2.05	0.65
1.08	0.81 - 1.43	0.62
1.05	0.76 - 1.42	0.78
0.94	0.67 - 1.31	0.71
0.71	0.34 - 1.36	0.31
0.83	0.60 - 1.15	0.25

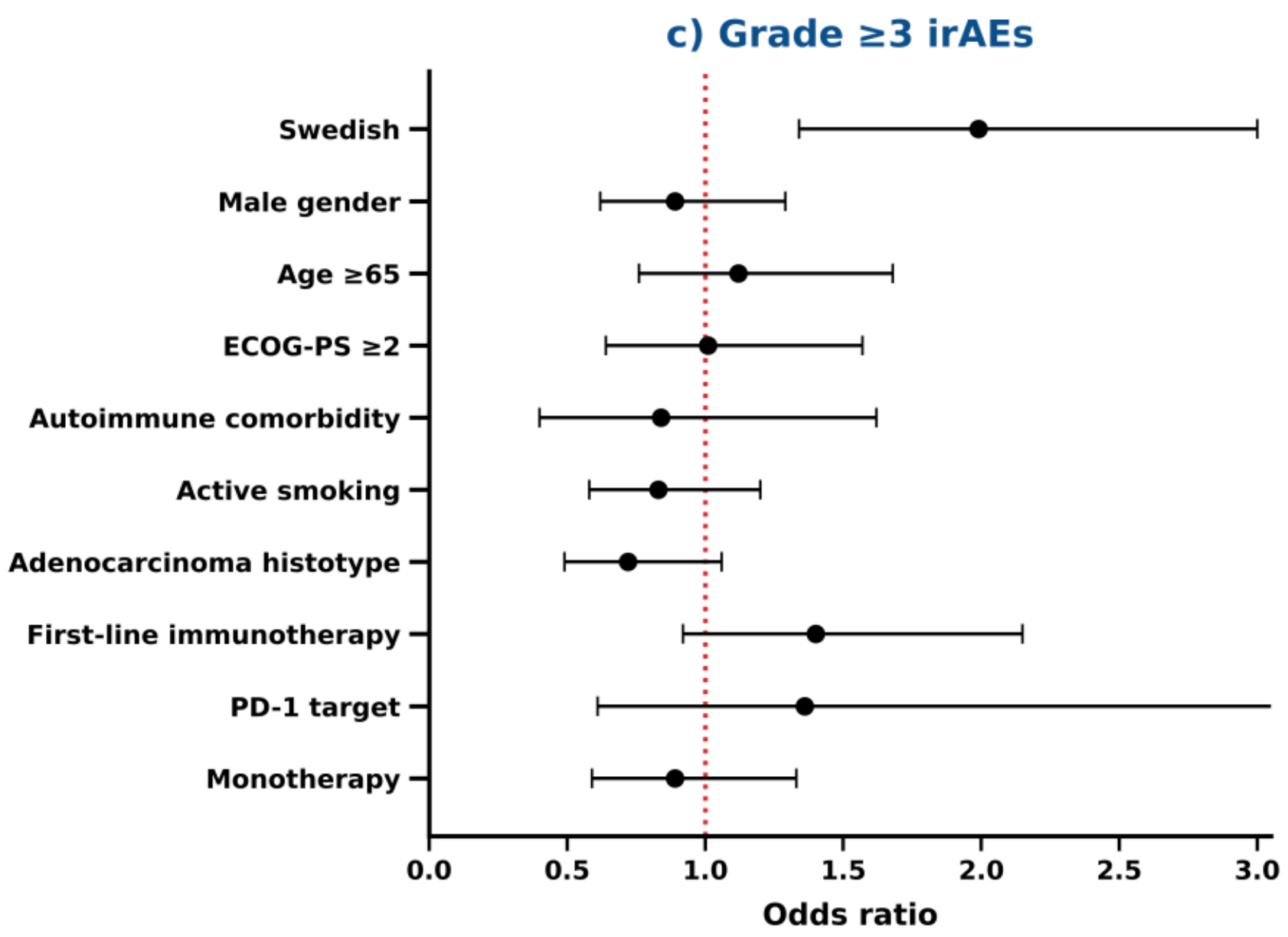

OR	95% CI	*p*-value
1.99	1.34 - 3.00	<0.001
0.89	0.62 - 1.29	0.54
1.12	0.76 - 1.68	0.57
1.01	0.64 - 1.57	0.95
0.84	0.40 - 1.62	0.63
0.83	0.58 - 1.20	0.32
0.72	0.49 - 1.06	0.10
1.40	0.92 - 2.15	0.11
1.36	0.61 - 3.65	0.48
0.89	0.59 - 1.33	0.56
