# Supplementary material for: Real-world comparative evaluation of immune-related toxicities in non-small cell lung cancer patients treated with immune-checkpoint inhibitors in Greece and Sweden
Source: Front Immunol. 2026 Aug 10;17:1904934. doi: 10.3389/fimmu.2026.1904934 (PMC13500524; doi:10.3389/fimmu.2026.1904934)
Supplement: Supplementary file 3 [file DataSheet1.docx]

**Supplementary material**

**Supplementary Table S1. Distribution of genetic alterations in the two cohorts**

| **Genetic alterations** | **Greek cohort (%)** | **Swedish cohort (%)** | ***p*-value** |
| --- | --- | --- | --- |
| Patients | 653 (100.0) | 678 (100.0) |  |
| Molecular profiling | 441 (67.5) | 611 (90.1) | <0.0001 |
| KRAS | 80 (12.3) | 210 (34.4) | <0.0001 |
| EGFR | 9 (1.4) | 21 (3.4) | 0.18 |
| BRAF | 9 (1.4) | 24 (3.9) | 0.08 |
| ALK (IHC / FISH) | 8 (1.2) | 8 (1.3) | 0.51 |
| ERBB2 | 5 (0.8) | 4 (0.7) | 0.53 |
| RET | 4 (0.6) | 5 (0.8) | >0.99 |
| MET (exon 14 skipping or amplification) | 10 (1.5) | 26 (4.3) | 0.08 |
| ROS1 (IHC / FISH) | 1 (0.2) | 1 (0.2) | >0.99 |
| TP53 | 36 (5.5) | 24 (3.9) | <0.01 |
| STK11 | 11 (1.7) | 0 (0) | <0.0001 |

IHC: Immunohistochemistry; FISH: Fluorescence in situ hybridization.

**Additional adjusted analysis**

Expanded multivariable logistic regression models were constructed for three prespecified outcomes: (i) overall irAE occurrence, (ii) multiple irAEs, and (iii) severe grade ≥3 irAEs. Models included cohort, sex, age, ECOG-PS, autoimmune comorbidity, current smoking status, histological subtype, treatment line, ICI target, and immunotherapy regimen. In this expanded analysis, the Swedish cohort remained independently associated with severe grade ≥3 irAEs (OR: 1.99, 95%CI: 1.34–3.00; *p*<0.001), while cohort was not independently associated with overall irAE occurrence or multiple irAEs. Model calibration was assessed using the Hosmer–Lemeshow goodness-of-fit test, which did not indicate statistically significant lack of fit for overall irAEs, multiple irAEs, or grade ≥3 irAEs.

**Supplementary Table S2. Multivariable analysis for occurrence i) overall occurrence of irAEs, ii) incidence of multiple irAEs, and iii) incidence of severe (Grade≥3) irAEs.**

| **i) Overall irAEs occurrence** | | | |
| --- | --- | --- | --- |
| **Parameters** | **OR** | **95% CI** | ***p*-value** |
| Swedish cohort | 0.88 | 0.69 – 1.14 | 0.34 |
| Male gender | 1.02 | 0.80 – 1.31 | 0.85 |
| Age ≥65 years | 1.07 | 0.84 – 1.38 | 0.57 |
| ECOG-PS ≥2 | 1.06 | 0.80 – 1.39 | 0.70 |
| Autoimmune comorbidity | 1.18 | 0.74 – 1.87 | 0.49 |
| Current smoking status | 1.09 | 0.86 – 1.38 | 0.49 |
| Adenocarcinoma histotype | 1.26 | 0.97 – 1.64 | 0.09 |
| 1^st^ line immunotherapy | 0.84 | 0.64 – 1.12 | 0.24 |
| PD-1 target | 0.65 | 0.38 – 1.10 | 0.11 |
| Monotherapy | 0.86 | 0.66 – 1.14 | 0.29 |
| **ii) Multiple irAEs** | | | |
| **Parameters** | **OR** | **95% CI** | ***p*-value** |
| Swedish cohort | 0.98 | 0.73 – 1.32 | 0.91 |
| Male gender | 0.93 | 0.69 – 1.24 | 0.60 |
| Age ≥65 years | 0.85 | 0.63 – 1.15 | 0.29 |
| ECOG-PS ≥2 | 1.18 | 0.85 – 1.67 | 0.32 |
| Autoimmune comorbidity | 1.14 | 0.66 – 2.05 | 0.65 |
| Current smoking status | 1.08 | 0.81 – 1.43 | 0.62 |
| Adenocarcinoma histotype | 1.05 | 0.76 – 1.42 | 0.78 |
| 1^st^ line immunotherapy | 0.94 | 0.67 – 1.31 | 0.71 |
| PD-1 target | 0.71 | 0.34 – 1.36 | 0.31 |
| Monotherapy | 0.83 | 0.60 – 1.15 | 0.25 |
| **iii) Severe (Grade≥3) irAEs** | | | |
| **Parameters** | **OR** | **95% CI** | ***p*-value** |
| Swedish cohort | 1.99 | 1.34 – 3.00 | <0.001 |
| Male gender | 0.89 | 0.62 – 1.29 | 0.54 |
| Age ≥65 years | 1.12 | 0.76 – 1.68 | 0.57 |
| ECOG-PS ≥2 | 1.01 | 0.64 – 1.57 | 0.95 |
| Autoimmune comorbidity | 0.84 | 0.40 – 1.62 | 0.63 |
| Current smoking status | 0.83 | 0.58 – 1.20 | 0.32 |
| Adenocarcinoma histotype | 0.72 | 0.49 – 1.06 | 0.10 |
| 1^st^ line immunotherapy | 1.40 | 0.92 – 2.15 | 0.11 |
| PD-1 target | 1.36 | 0.61 – 3.65 | 0.48 |
| Monotherapy | 0.89 | 0.59 – 1.33 | 0.56 |

Hosmer–Lemeshow goodness-of-fit p-values were: overall irAEs, p=0.826; multiple irAEs, p=0.499; grade ≥3 irAEs, p=0.052. CI: Confidence interval; ECOG-PS: Eastern Cooperative Oncology Group-performance status; irAEs: Immune-related adverse events; OR: Odds ratio; PD-1: Programmed-death receptor-1.

**Supplementary Figure Captions**

Supplementary Figure S1. Predominant irAEs per line of treatment in the Greek and Swedish cohorts. Shifts in the type of predominant irAEs were observed per line of treatment in both cohorts. In the Greek cohort, the most frequently reported irAEs during first-line treatment were dermatologic events (21.3%), endocrine toxicities (17.9%), and colitis (10.7%). In contrast, among patients treated in second-line or beyond, dermatologic and endocrine toxicities became even more prevalent (29.4% and 22.5%, respectively), with pneumonitis (10.1%) emerging as the third most frequent irAE. In the Swedish cohort, the leading irAEs in the first-line setting were dermatologic toxicities (22.3%), followed by colitis (13.5%) and endocrine events (10.3%). In later-line treatments, dermatologic (18.3%) and endocrine (17.3%) toxicities remained among the most frequent, while hepatitis (10.3%) became the third most common event. L: line; irAEs: immune-related adverse events.

Supplementary Figure S2. Expanded multivariable logistic regression sensitivity analysis for irAE outcomes. Adjusted odds ratios (OR) are shown for (A) overall irAE occurrence, (B) multiple irAEs, and (C) severe grade ≥3 irAEs. Models included cohort, sex, age, ECOG-PS, autoimmune comorbidity, current smoking status, histological subtype, treatment line, ICI target, and immunotherapy regimen. The Swedish cohort remained independently associated with severe grade ≥3 irAEs but not with overall irAE occurrence or multiple irAEs. CI: confidence interval; ECOG-PS: Eastern Cooperative Oncology Group performance status; ICI: immune checkpoint inhibitor; irAE: immune-related adverse event; OR: Odds ratio; PD-1: Programmed-death receptor-1.
