# Supplementary material for: Real-world comparative evaluation of immune-related toxicities in non-small cell lung cancer patients treated with immune-checkpoint inhibitors in Greece and Sweden
Source: Front Immunol. 2026 Aug 10;17:1904934. doi: 10.3389/fimmu.2026.1904934 (PMC13500524; doi:10.3389/fimmu.2026.1904934)
Supplement: Supplementary file 4 [file Table1.docx]

STROBE Statement—checklist of items that should be included in reports of observational studies

|  | | | Item No. | Recommendation | Page  No. | | | Relevant text from manuscript |
| --- | --- | --- | --- | --- | --- | --- | --- | --- |
| **Title and abstract** | | | 1 | (*a*) Indicate the study’s design with a commonly used term in the title or the abstract | 1 | | | Title indicates real-world comparative evaluation of irAEs in NSCLC cohorts (Greece vs Sweden). |
|  |  |  |  | (*b*) Provide in the abstract an informative and balanced summary of what was done and what was found | 2 | | | Abstract summarizes background, methods, key results and conclusion with effect sizes. |
| Introduction | | | | | | | |  |
| Background/rationale | | | 2 | Explain the scientific background and rationale for the investigation being reported | 3-4 | | | Introduction provides scientific background/rationale on irAEs and geoenvironmental/host factors. |
| Objectives | | | 3 | State specific objectives, including any prespecified hypotheses | 4 | | | To address the limited evidence on geoepidemiologic patterns of immunotherapy toxicity and to provide insights into potential population-level modifiers of irAE risk. |
| Methods | | | | | | | |  |
| Study design | | | 4 | Present key elements of study design early in the paper | 4-5 | | | Section 2.1 Study population and data sources. Study design: retrospective comparative cohort study; harmonized dataset. |
| Setting | | | 5 | Describe the setting, locations, and relevant dates, including periods of recruitment, exposure, follow-up, and data collection | 4-5 | | | Section 2.1 Study population and data sources. Setting and dates: Athens (2014–2023) and Stockholm (2016–2022) thoracic oncology centers. |
| Participants | | | 6 | (*a*) *Cohort study*—Give the eligibility criteria, and the sources and methods of selection of participants. Describe methods of follow-up  *Case-control study*—Give the eligibility criteria, and the sources and methods of case ascertainment and control selection. Give the rationale for the choice of cases and controls  *Cross-sectional study*—Give the eligibility criteria, and the sources and methods of selection of participants | 5, Figure 1 (Flow chart) | | | Section 2.1 Study population and data sources. Eligibility: at least one dose of ICIs for advanced/metastatic NSCLC. Exclusions: early-stage, dual ICI, special histologies. |
|  |  |  |  | (*b*) *Cohort study*—For matched studies, give matching criteria and number of exposed and unexposed  *Case-control study*—For matched studies, give matching criteria and the number of controls per case | 5 | | | Recruitment: consecutive/all-comers advanced NSCLC receiving ≥1 ICI dose at each center. |
| Variables | | | 7 | Clearly define all outcomes, exposures, predictors, potential confounders, and effect modifiers. Give diagnostic criteria, if applicable | 5-6 | | | Sections 2.2 Data collections and 2.3 Outcomes.  Variables: baseline demographics/clinical, treatment factors, irAE outcomes and grading (CTCAE v5.0). |
| Data sources/ measurement | | | 8* | For each variable of interest, give sources of data and details of methods of assessment (measurement). Describe comparability of assessment methods if there is more than one group | 6 | | | Section 2.2 Data collection. Data sources/measurement: records abstraction, harmonization, predefined organ categories, CTCAE v5.0*.* |
| Bias | | | 9 | Describe any efforts to address potential sources of bias | 6, 9, 13, Supplementary Material, Supplementary Table S2, Supplementary Figure S2 | | | Statistical analysis, Limitation section in the discussion. Retrospective design, single-center, residual confounding, competing risks discussed. Additional adjusted analysis in Supplementary Material. |
| Study size | | | 10 | Explain how the study size was arrived at | 4, 7, Figure 1 | | | Study size: 1,331 patients included (653 Greece; 678 Sweden). |
| Quantitative variables | | 11 | | Explain how quantitative variables were handled in the analyses. If applicable, describe which groupings were chosen and why | 6, 18-20, Tables 1 and 2, Supplementary Material, Supplementary Table S2 | | Section 2.5 Statistical analysis. Continuous variables were assessed for normality using the Shapiro–Wilk test and summarized as medians (range) when non-normally distributed; groups were compared with the Mann–Whitney U test. Categorical variables were summarized as n (%) and compared using *χ²* or Fisher’s exact tests. For multivariable logistic regression, outcomes were modeled as binary variables (any irAE, multisystem irAEs, and grade ≥3 irAEs), with prespecified covariates selected based on clinical relevance and baseline between-cohort imbalances | |
| Statistical methods | | 12 | | (*a*) Describe all statistical methods, including those used to control for confounding | 6-7 | | Section 2.5 Statistical analysis. Statistical methods for outcomes: *χ²*/Fisher; Shapiro–Wilk; Mann–Whitney; logistic regression. | |
|  |  |  |  | (*b*) Describe any methods used to examine subgroups and interactions | 6-7 | | Subgroup analyses were descriptive/stratified by line of therapy, PD-L1 expression, and treatment regimen; no formal interaction tests were performed. | |
|  |  |  |  | (*c*) Explain how missing data were addressed | 5, 13  Figure 1 | | Section 2.1 Study population and data sources. Cases with missing or insufficient data on toxicity outcomes or treatment characteristics were not considered for analysis; minimal exclusions noted. | |
|  |  |  |  | (*d*) *Cohort study*—If applicable, explain how loss to follow-up was addressed  *Case-control study*—If applicable, explain how matching of cases and controls was addressed  *Cross-sectional study*—If applicable, describe analytical methods taking account of sampling strategy | 5 | | Not applicable; retrospective toxicity documentation. Cases with missing data were excluded. However, missing data exclusions were minimal (21/674 in the Greek cohort; none in the Swedish cohort). | |
|  |  |  |  | (*e*) Describe any sensitivity analyses |  | | Not applicable | |
| Results | | | | | | | | |
| Participants | | 13* | | (a) Report numbers of individuals at each stage of study—eg numbers potentially eligible, examined for eligibility, confirmed eligible, included in the study, completing follow-up, and analysed | 5, 7,  Figure 1 (Flow chart) | | Participant numbers at each stage: inclusion/exclusion and Figure 1 flow diagram. | |
|  |  |  |  | (b) Give reasons for non-participation at each stage | Figure 1 | | Reasons for non-participation: exclusions due to criteria/insufficient data; summarized in Figure 1. | |
|  |  |  |  | (c) Consider use of a flow diagram | Figure 1 | | Figure 1 referenced in Methods. | |
| Descriptive data | | 14* | | (a) Give characteristics of study participants (eg demographic, clinical, social) and information on exposures and potential confounders | 7-8, 18-19 (Table 1, Supplementary Table S1, Supplementary Table S2 | | Descriptive data: cohort characteristics and treatment patterns in Table 1 and in the section 3.1 Baseline clinical and pathological characteristics. | |
|  |  |  |  | (b) Indicate number of participants with missing data for each variable of interest | 13, Figure 1 | | Missing data: minimal missing data exclusions (21/674 Greece; none Sweden) in limitations. | |
|  |  |  |  | (c) *Cohort study*—Summarise follow-up time (eg, average and total amount) | 7, 18-19  (Table 1) | | Follow-up time: median immunotherapy exposure time reported and compared (Table 1). | |
| Outcome data | | 15* | | *Cohort study*—Report numbers of outcome events or summary measures over time | 7-9, 19-20 (Table 2), Figures 2 and 3 | | Outcome data: irAE incidence, grade≥3, multisystem, organ-specific distribution (Table 2; Figures 2 and 3). | |
|  |  |  |  | *Case-control study—*Report numbers in each exposure category, or summary measures of exposure |  | | Not applicable | |
|  |  |  |  | *Cross-sectional study—*Report numbers of outcome events or summary measures |  | | Not applicable | |
| Main results | | 16 | | (*a*) Give unadjusted estimates and, if applicable, confounder-adjusted estimates and their precision (eg, 95% confidence interval). Make clear which confounders were adjusted for and why they were included | Figure 4, Supplementary Figure S2 | | Main results in Figure 4 and Supplementary Figure S2: Multivariable logistic regression analyses showing adjusted OR for (a) occurrence of any irAE, (b) occurrence of multiple irAEs, and (c) occurrence of grade ≥3 irAEs. | |
|  |  |  |  | (*b*) Report category boundaries when continuous variables were categorized | 7-9, 18-20, Tables 1 and 2, Figure 4, Supplementary Figure S2 | | Continuous variables were categorized using prespecified cutoffs: age ≥65 years; ECOG-PS ≥2; PD-L1 TPS >1% (positive) and ≥50% (high). | |
|  |  |  |  | (*c*) If relevant, consider translating estimates of relative risk into absolute risk for a meaningful time period |  | | Not applicable: outcomes were reported as proportions and OR (logistic regression). | |
| Other analyses | 17 | | Report other analyses done—eg analyses of subgroups and interactions, and sensitivity analyses | | 18-20, Tables 1 and 2, Figure 2, Figure 4, Supplementary Material, Supplementary Table S2, Supplementary Figure S1, Supplementary Figure S2 | Outcomes were additionally examined in predefined subgroups by line of therapy (1L vs ≥2L), PD-L1 category (<50% vs ≥50%), and regimen (1L monotherapy vs chemo-immunotherapy). Covariate-adjusted associations were evaluated using multivariable logistic regression for any irAE, multiple irAEs, and grade ≥3 irAEs (Figure 4). No formal interaction tests or sensitivity analyses were performed. Additional adjusted analysis in Supplementary material. | | |
| Discussion | | | | | | | | |
| Key results | 18 | | Summarise key results with reference to study objectives | | 9-13 | Key results summarized in Discussion: higher severe irAEs and higher hepatitis/nephritis in the Swedish cohort | | |
| Limitations | 19 | | Discuss limitations of the study, taking into account sources of potential bias or imprecision. Discuss both direction and magnitude of any potential bias | | 13 | Limitations: retrospective grading, single-center, confounding, competing risks. | | |
| Interpretation | 20 | | Give a cautious overall interpretation of results considering objectives, limitations, multiplicity of analyses, results from similar studies, and other relevant evidence | | 9-13 | Interpretation: latitude-linked autoimmunity, UV/vitamin D, microbiome, implications for surveillance. | | |
| Generalisability | 21 | | Discuss the generalisability (external validity) of the study results | | 12-13 | Generalisability: limited; calls for prospective multicenter validation and mechanistic data. | | |
| Other information | | |  | | | | | |
| Funding | 22 | | Give the source of funding and the role of the funders for the present study and, if applicable, for the original study on which the present article is based | | 14-15 | See funding section. The funders had no role in the collection, the interpretation of data, or the writing of the manuscript. | | |

CTCAEv5.0: common terminology criteria for adverse events version 5; ICI: immune checkpoint inhibitors; irAE: immunotherapy-related adverse events; L: line; NSCLC: non-small cell lung cancer; OR: odds ratios; vs: versus

*Give information separately for cases and controls in case-control studies and, if applicable, for exposed and unexposed groups in cohort and cross-sectional studies.

**Note:** An Explanation and Elaboration article discusses each checklist item and gives methodological background and published examples of transparent reporting. The STROBE checklist is best used in conjunction with this article (freely available on the Web sites of PLoS Medicine at http://www.plosmedicine.org/, Annals of Internal Medicine at http://www.annals.org/, and Epidemiology at http://www.epidem.com/). Information on the STROBE Initiative is available at www.strobe-statement.org.
